# Upper-leaf architecture was associated with grain yield variation in an *indica–japonica* rice recombinant inbred line population

**DOI:** 10.3389/fpls.2026.1873410

**Published:** 2026-07-10

**Authors:** Xuetong Zhu, Jian Liu, Jing Yang, Yikai Ding, Mengya Li, Xin Lu, Fei Zhao

**Affiliations:** Tianjin Agricultural University, Tianjin, China

**Keywords:** flag leaf morphology, grain yield, *indica–japonica* RIL population, phenotypic selection, rice, SPAD dynamics

## Abstract

Rice yield improvement depends on the coordinated optimization of plant architecture and physiological performance. In this study, an F8 *indica–japonica* recombinant inbred line (RIL) population derived from ‘Zhongyouzao8’ × ‘Toyonishiki’ was evaluated in a single field environment to examine how heading progression, flag-leaf SPAD dynamics around heading, and upper-leaf architecture were associated with grain yield. A total of 159 RILs and the two parents were phenotyped for grain yield, initial heading date, full heading date, pre-heading SPAD (SPAD1), post-heading SPAD at the onset of grain filling (SPAD2), flag leaf angle, plant height, and flag leaf traits. Substantial phenotypic variation and evident transgressive segregation were observed for most traits. Grain yield ranged from 2.62 to 9.88 t ha^-^¹, and several traits showed moderate to high broad-sense heritability. Correlation, regression, principal component analysis, and high- versus low-yielding group comparisons suggested that, under the conditions evaluated, grain yield variation was more closely associated with upper-leaf architecture than with heading progression itself, whereas SPAD dynamics provided complementary but weaker information. However, the regression model explained only a limited proportion of yield variability, indicating that additional unmeasured yield components may also have contributed substantially to grain yield. Several preliminary candidate lines carrying relatively high grain yield and favorable combinations of flag leaf morphology and SPAD-related phenotypic profiles were identified from the single-environment screening. These findings are exploratory and require multi-environment validation before these trait combinations can be used as stable selection criteria in rice breeding.

## 1 Introduction

Rice (*Oryza sativa* L.) is a staple food for more than half of the world’s population, and sustained improvement in rice productivity remains a major objective of crop breeding and production research (Fukagawa and Ziska, 2019). However, further yield gains increasingly depend on ideotype design and on the coordinated optimization of morphological and physiological traits rather than on the improvement of single agronomic traits in isolation (Peng et al., 2008; Li et al., 2025b). In rice, canopy architecture, leaf morphology, and reproductive-stage source activity jointly influence light distribution, canopy photosynthesis, grain filling, and final productivity (Burgess et al., 2017; Pan et al., 2023; Yagioka et al., 2025).

Among the breeding materials available for dissecting favorable yield-related trait combinations, recombinant inbred line (RIL) populations derived from *indica–japonica* crosses are especially valuable. The *indica* and *japonica* subspecies harbor substantial genetic divergence and complementary favorable alleles, and their recombination can generate broad phenotypic segregation and transgressive variation for agronomic traits in derived progeny (Marathi et al., 2012; Bai et al., 2011; Zhang et al., 2014). Previous studies have also reported that *indica–japonica* hybrid materials can show heterosis, increased yield potential, and enhanced stress-tolerance-related performance compared with more closely related backgrounds or parental lines, indicating that such inter-subspecific crosses can provide useful genetic resources for breeding and phenotypic dissection (Shahid et al., 2011; Sun et al., 2025). Because RILs are genetically fixed and can be repeatedly evaluated across traits and environments, they are particularly suitable for phenotypic dissection, candidate line identification, and breeding-oriented trait integration (Bai et al., 2011; Ata-Ul-Karim et al., 2022).

Within such populations, heading progression, flag-leaf SPAD dynamics around heading, and upper-leaf architecture are three trait modules likely to contribute jointly to grain yield variation. Heading date is a key determinant of rice adaptation and yield formation because it affects the coordination between crop development and environmental conditions, thereby influencing grain-filling duration and yield potential (Yano et al., 2000; Xue et al., 2008; Sun et al., 2023; Su et al., 2025). Recent breeding studies further suggest that rice improvement should focus not simply on early or late heading, but on achieving an optimal heading-date window under specific ecological conditions (Su et al., 2025). Genetic analyses further indicate that heading-date gene combinations can exert pleiotropic effects on plant height and other agronomic traits (Zhou et al., 2016; Lee et al., 2024), and recent evidence also links flowering regulation with nitrogen-dependent developmental responses in rice (Yoshida et al., 2025). In parallel, SPAD-derived chlorophyll status provides a useful indicator of leaf functional dynamics across the transition from pre-heading to early grain filling. It has been widely used as a rapid and nondestructive indicator of leaf chlorophyll content and physiological status in rice (Takai et al., 2010; Kim and Kim, 2024), and variation in leaf color change during the later reproductive stage has been linked to grain filling, nitrogen remobilization, and late-stage N reuse dynamics (Tao et al., 2024; Xu et al., 2025). Upper-leaf architecture is another important component of rice yield potential. Traits such as flag leaf angle, length, width, and area directly affect light interception, canopy photosynthetic efficiency, and field population structure (Yue et al., 2006; Burgess et al., 2017). A favorable upper-leaf configuration has long been regarded as a key feature of ideal plant architecture in rice (Zhang et al., 2014; Huang et al., 2021; Zhu et al., 2020), and recent physiological and review studies further support the contribution of superior canopy morphology, NAL1-related leaf architecture, and source activity to stable biomass production and high yield performance (Pan et al., 2023; Velu et al., 2025; Yagioka et al., 2025).

Although substantial progress has been made in the genetic and physiological analysis of heading date, leaf functional traits, and upper-leaf architecture, previous studies have often addressed these dimensions separately, with emphasis on heading-date optimization (Xue et al., 2008; Sun et al., 2023; Su et al., 2025), flag leaf angle regulation (Huang et al., 2021; Li et al., 2025a), SPAD-related physiological variation (Takai et al., 2010; Kim and Kim, 2024; Tao et al., 2024; Xu et al., 2025), or specific elite/high-yielding cultivars (Pan et al., 2023; Yagioka et al., 2025), rather than on their joint phenotypic associations with yield. Comparatively less attention has been paid to the coordinated phenotypic relationships among heading progression, flag-leaf SPAD dynamics around heading, upper-leaf architecture, and grain yield within *indica–japonica* RIL populations. Given the broad phenotypic segregation and breeding value of such materials, a systematic phenotypic dissection of these trait dimensions may help identify favorable trait combinations associated with improved yield performance under specific field conditions. Therefore, in the present study, an *indica–japonica* rice RIL population was used to evaluate grain yield, initial heading date, full heading date, booting-stage SPAD before heading onset (SPAD1), post-heading SPAD at the onset of grain filling (SPAD2), flag leaf angle after full heading, flag leaf area, plant height, and flag leaf shape-related traits. The objectives of this study were to: (i) characterize the phenotypic variation of grain yield and related traits in the RIL population; (ii) clarify the phenotypic associations of grain yield with heading progression, SPAD dynamics, and upper-leaf architecture; and (iii) identify preliminary candidate lines with favorable phenotypic trait combinations for future validation in rice breeding.

## 2 Materials and methods

### 2.1 Plant materials

The *indica–japonica* recombinant inbred line (RIL) population used in this study was derived from a cross between the *indica* rice cultivar ‘Zhongyouzao8’ (ZYZ8) and the *japonica* rice cultivar ‘Toyonishiki’ (TK). This population had been previously developed and used for genetic analysis of rice quality-related traits (Yao et al., 2017), and the parental materials and the derived RIL population were provided by Shenyang Agricultural University. In the cross, ‘Zhongyouzao8’ was used as the female parent and ‘Toyonishiki’ as the male parent. The F1 plants were selfed to produce the F2 generation, and the population was subsequently advanced to the F8 generation by single-seed descent. These parental cultivars were selected because they represent *indica* and *japonica* backgrounds and showed contrasting phenotypic profiles in developmental timing, plant architecture, upper-leaf morphology, and grain yield-related performance, providing broad segregation for investigating yield-associated trait combinations. In the 2025 field experiment, 159 available RILs from this population, together with the two parental lines, were evaluated under field conditions for grain yield, heading-related traits, SPAD dynamics, flag leaf angle, plant height, and flag leaf morphological traits.

### 2.2 Field experiment and experimental design

The field experiment was conducted in 2025 at the Xiaozhan Rice Molecular Design Breeding Experimental Base, Baodi District, Tianjin, China. Seeds were sown on 25 May 2025, seedlings were transplanted on 20 June 2025, and plants were harvested on 21 October 2025, corresponding to the typical rice-growing season following wheat harvest in North China.

The experiment consisted of three independent replicated blocks, and the field layout was identical among blocks. Within each block, the materials were planted in numerical order to facilitate accurate line identification during repeated field phenotyping and harvest, and to reduce the risk of plot or sample mislabeling. Each plot consisted of three planted rows with 10 plants per row (3 rows × 10 plants). Plant spacing was 15 cm and row spacing was 30 cm. Border rows were established on both sides of the experimental field to ensure a uniform surrounding environment among plots. The effective harvested area of each plot was 1.35 m². Because the entries were not randomly assigned within blocks, potential field-position effects were considered in the interpretation of trait-yield relationships, and block effects were retained in the statistical analyses.

All plots were managed under uniform field conditions. Basal fertilizer was applied as a compound fertilizer (total nutrients ≥ 48%, N–P_2_O_5_–K_2_O = 24–14–10), and urea (total nutrients ≥ 46%) was top-dressed at the tillering stage. Nitrogen fertilizer was applied twice, as basal fertilizer and tillering fertilizer, at a ratio of 9:2. Irrigation and other field management practices followed local conventional rice cultivation procedures. The soil type of the experimental site was wet-cinnamon soil, and the soil total nitrogen content was 1.286 g kg^-^¹.

### 2.3 Trait measurement

#### 2.3.1 Grain yield

Grain yield was determined on a plot basis. At maturity, all 30 plants in each plot were harvested and threshed together, and grain weight was recorded at 14% moisture content. Grain yield was converted according to Equation 1:

(1) $$Grain\;yield\;(t\;ha^{-1})=[grain\;weight\;per\;plot\;(kg)/\;1.35]\times 10$$

where 1.35 is the effective harvested area (m²) of each plot.

#### 2.3.2 Heading-related traits

Heading was defined as the stage when the middle part of the young panicle had emerged from the flag leaf sheath, following the Standard Evaluation System for Rice (IRRI, 2013). Initial heading date was recorded as the date on which the first plant in a plot headed, whereas full heading date was recorded as the date on which approximately 90% of the plants in a plot had headed. Both heading dates were converted to days after transplanting (DAT) for analysis, as commonly used in transplanted rice studies (Lee et al., 2021).

#### 2.3.3 SPAD measurements

SPAD values were measured twice on the flag leaf using a TYS-4N plant nutrition meter (Top Cloud-Agri, China). The first measurement (SPAD1) was obtained at the booting stage, when all lines remained pre-heading but were close to heading. The second measurement (SPAD2) was obtained after all lines had reached full heading, at the onset of grain filling. Readings were taken at the middle part of the flag leaf while avoiding direct sunlight and the midrib.

Six plants were measured in each plot at each sampling time, and the mean of the sampled plants was used as the plot value.

#### 2.3.4 Flag leaf angle, flag leaf traits, and plant height

Flag leaf angle was measured using a TPS-BX-1 rice phenotyping system (Top Cloud-Agri, China). Measurements were conducted on 19 September 2025, after all lines had reached full heading. The measured trait was the angle between the flag leaf and the culm. Five plants were measured per plot, and the mean value was used as the plot value.

Flag leaf area, length, and width were measured non-destructively using a YMJ-B portable leaf area meter (Top Cloud-Agri, China) on the same day. Before measurement, the instrument was calibrated using the standard reference leaf template provided by the manufacturer. During measurement, the intact flag leaf was gently clamped and scanned along the leaf blade without excision or removal from the plant. Three plants were measured in each plot, and the mean of the sampled plants was used as the plot value. Because all flag leaf measurements were non-destructive, the measured plants remained intact and were included in the subsequent plot-level grain yield determination.

Plant height was measured non-destructively on the same day and was defined as the distance from the ground surface to the top of the panicle, excluding awns. Five plants were measured per plot, and the mean value was used as the plot value.

Trait-specific sampling numbers were used following common field-based rice phenotyping practice, in which representative plants are sampled within each plot and the sampling intensity is adjusted according to the nature, throughput, and workload of each measurement. Grain yield was determined from all 30 plants in each plot to obtain a plot-level yield value. For non-destructive measurements, SPAD was measured on six plants to better represent within-plot variation in leaf physiological status; flag leaf angle and plant height were measured on five plants as rapid architectural measurements; and flag leaf area, length, and width were measured on three plants because each intact flag leaf had to be individually clamped and scanned with the portable leaf area meter, which was more time-consuming than SPAD or angle measurements. For all traits measured on sampled plants, plant-level values were averaged to obtain plot-level means for statistical analysis, and all sampled plants remained included in the final plot-level grain yield determination.

### 2.4 Derived variables

Several derived variables were calculated to characterize heading progression, flag-leaf SPAD dynamics around heading, and flag leaf morphology.

Heading interval, SPAD change, SPAD change rate, and flag leaf length/width ratio were calculated using Equations 2–5.

(2) HI = FHD – IHD

where FHD is full heading date and IHD is initial heading date, both recorded as days after transplanting (DAT); HI is expressed in days.

SPAD change (SC) was calculated as:

(3) SC = SPAD2 − SPAD1

SPAD change rate (SCR) was calculated as:

(4) SCR (%)=[(SPAD2 − SPAD1)/SPAD1]×100

Flag leaf length/width ratio (FLR) was calculated as:

(5) FLR = FLL / FLW

where FLL is flag leaf length (cm) and FLW is flag leaf width (cm); FLR is dimensionless. SC is expressed in SPAD units, SCR is expressed as a percentage (%), and grain yield is expressed as t ha^-^¹.

### 2.5 Statistical analysis

For grain yield and heading-related traits, plot-based data from the three replicated blocks were used directly for statistical analysis. For SPAD, flag leaf angle, plant height, and flag leaf traits, the mean of the sampled plants within each plot was first calculated and then used as the plot value. All statistical analyses and figure preparation were performed in R software (version 4.5.2; R Core Team, Vienna, Austria) using base functions and the packages readxl, dplyr, tidyr, stringr, ggplot2, patchwork, scales, forcats, and openxlsx.

Descriptive statistics, including mean, standard deviation (SD), range, and coefficient of variation (CV), were calculated for each trait based on genotype means across the three replicates. The coefficient of variation, ANOVA model, broad-sense heritability, and composite score were calculated using Equations 6–9:

(6) $$CV\;(\%)\;=\;(SD\;/\;\bar{x})\;\times \;100$$

Phenotypic variation among genotypes was evaluated using analysis of variance according to the following linear model:

(7) $$Y_{ij}=\mu +G_{i}+B_{j}+\epsilon_{ij}$$

where Y_ij_ is the observed value of genotype i in block j, μ is the overall mean, G_i_ is the effect of genotype i, B_j_ is the effect of block j, and ϵ_ij_ is the residual error.

Broad-sense heritability on a line-mean basis was estimated as:

(8) $$H^{2}=\sigma g^{2}/(\sigma g^{2}+\sigma e^{2}/r)$$

where σg² is the genotypic variance, σe² is the residual variance, and r is the number of replicates.

Frequency distributions were examined for all measured traits to characterize phenotypic segregation in the RIL population. Transgressive segregation was evaluated by comparing the trait distribution of the RILs with the parental range. RILs with values exceeding the higher parent or lower than the lower parent were regarded as transgressive lines.

Pearson’s correlation coefficients were calculated among traits using genotype means across the three replicates. The correlation matrix was used as an exploratory phenotypic association analysis; therefore, unadjusted P values were reported to indicate nominal significance levels, and no multiple-testing correction was applied. To identify the main phenotypic factors associated with grain yield, multiple linear regression analysis was performed with grain yield as the response variable and full heading date, SPAD change rate, flag leaf angle, plant height, and flag leaf length as explanatory variables. Collinearity among explanatory variables was examined before model fitting, and variables showing strong redundancy were not included simultaneously in the final model. Standardized regression coefficients were calculated by refitting the final model after z-score standardization of both grain yield and the explanatory variables.

Principal component analysis (PCA) was performed using standardized genotype means for six representative variables selected to capture the three major phenotypic modules emphasized in this study while limiting redundancy among closely related traits: initial heading date (IHD) for heading progression; SPAD2 and SPAD change rate (SCR) for flag-leaf chlorophyll status and its change around heading; and flag leaf length, flag leaf area, and flag leaf length/width ratio (FLR) for upper-leaf architecture. The six-variable set was selected because it represented the main biological modules of interest and avoided over-weighting highly correlated measurements in the PCA. Other measured traits were evaluated in the correlation, regression, and group-comparison analyses but were not included in the PCA biplot to maintain a concise and non-redundant multivariate summary. In addition, the 159 RILs were ranked according to mean grain yield across the three replicates. The top 20% of lines (32 RILs) were defined as the high-yielding group, and the bottom 20% of lines (32 RILs) were defined as the low-yielding group. Differences in key phenotypic traits between the two groups were compared using Welch’s two-sample t-test. The two parents were included as references in trait distribution analysis but were not included in the high-yielding and low-yielding group classification.

Based on grain yield and multivariate phenotypic performance, preliminary candidate lines with favorable phenotypic combinations were further identified within the high-yielding subset. A composite score was calculated from six equally weighted standardized components: grain yield (GY), flag leaf length (FLL), flag leaf length/width ratio (FLR), leaf angle (LA), inverse plant height (−PH), and inverse SPAD change rate (−SCR). Because grain yield was included both in the initial top-yielding subset selection and in the composite score, the selected lines should be regarded as preliminary candidates from a single-environment phenotypic screening rather than as independently validated stable high-yielding lines. The composite score was defined as:

(9) Composite score=[z(GY)+z(FLL)+z(FLR)+z(LA)+z(−PH)+z(−SCR)]/6

where z indicates the standardized value of each trait. Negative signs for PH and SCR indicate that lower plant height and lower SPAD change rate contributed positively to the final ranking.

## 3 Results

### 3.1 Phenotypic variation and genetic parameters of grain yield and related traits in the RIL population

Substantial phenotypic variation was observed for grain yield and all measured agronomic and leaf-related traits in the *indica–japonica* RIL population (**Table 1**). Grain yield ranged from 2.62 to 9.88 t ha^-^¹ with a population mean of 6.18 t ha^-^¹, whereas full heading date varied from 54.00 to 73.00 DAT. Plant height, leaf angle, and flag leaf area also showed broad variation, ranging from 108.81 to 172.95 cm, 8.27 to 90.77°, and 15.26 to 53.26 cm², respectively. Analysis of variance revealed significant genotypic effects for most traits, whereas SPAD change and SPAD change rate showed comparatively weak genotypic differentiation.

**Table 1:** Descriptive statistics and broad-sense heritability of grain yield and related traits in the *indica–japonica* RIL population.

Trait	Unit	ZYZ8	TK	Mean ± SD	Range	CV (%)	H²
Grain yield	t ha^-^¹	6.05	5.53	6.18 ± 1.32	2.62–9.88	21.36	0.725
Initial heading date	DAT	57.00	51.00	57.07 ± 3.67	48.67–67.67	6.42	0.898
Full heading date	DAT	60.67	54.67	64.21 ± 4.15	54.00–73.00	6.46	0.894
Heading interval	d	3.67	3.67	7.14 ± 1.54	2.00–15.67	21.52	0.478
SPAD1	–	35.41	42.18	36.54 ± 2.30	30.84–40.93	6.29	0.613
SPAD2	–	38.36	44.12	39.80 ± 2.58	32.43–45.79	6.47	0.718
SPAD change	–	2.96	1.94	3.26 ± 2.12	-2.36–9.67		0.175
SPAD change rate	%	8.37	5.06	9.48 ± 6.13	-5.44–28.54		0.154
Flag leaf angle	°	43.54	22.93	30.53 ± 15.84	8.27–90.77	51.87	0.727
Plant height	cm	150.44	108.89	141.64 ± 10.87	108.81–172.95	7.67	0.918
Flag leaf length	cm	31.35	25.13	26.35 ± 3.97	14.90–36.83	15.08	0.411
Flag leaf width	cm	1.49	1.28	1.49 ± 0.21	1.08–2.11	14.15	0.892
Flag leaf area	cm²	31.38	23.91	30.51 ± 6.91	15.26–53.26	22.64	0.686
Flag leaf length/width ratio	–	21.00	19.71	18.00 ± 3.13	11.41–28.12	17.37	0.544

SPAD1, booting-stage SPAD before heading onset; SPAD2, post-heading SPAD at the onset of grain filling; DAT, days after transplanting; CV, coefficient of variation; H², broad-sense heritability on a line-mean basis. CV values are not shown for signed derived traits (SPAD change and SPAD change rate).

Broad-sense heritability estimates were generally moderate to high, although the magnitude varied among traits (**Table 1**). The highest heritability values were detected for plant height (H² = 0.918), initial heading date (0.898), full heading date (0.894), and flag leaf width (0.892), followed by leaf angle (0.727), grain yield (0.725), and flag leaf area (0.686). In contrast, heading interval (0.478), flag leaf length (0.411), and SPAD change-related traits (0.154–0.175) showed relatively lower heritability. These results indicate that most measured traits, especially heading date, plant height, and upper-leaf architectural traits, were under substantial genetic control in this RIL population.

**Figure 1:**
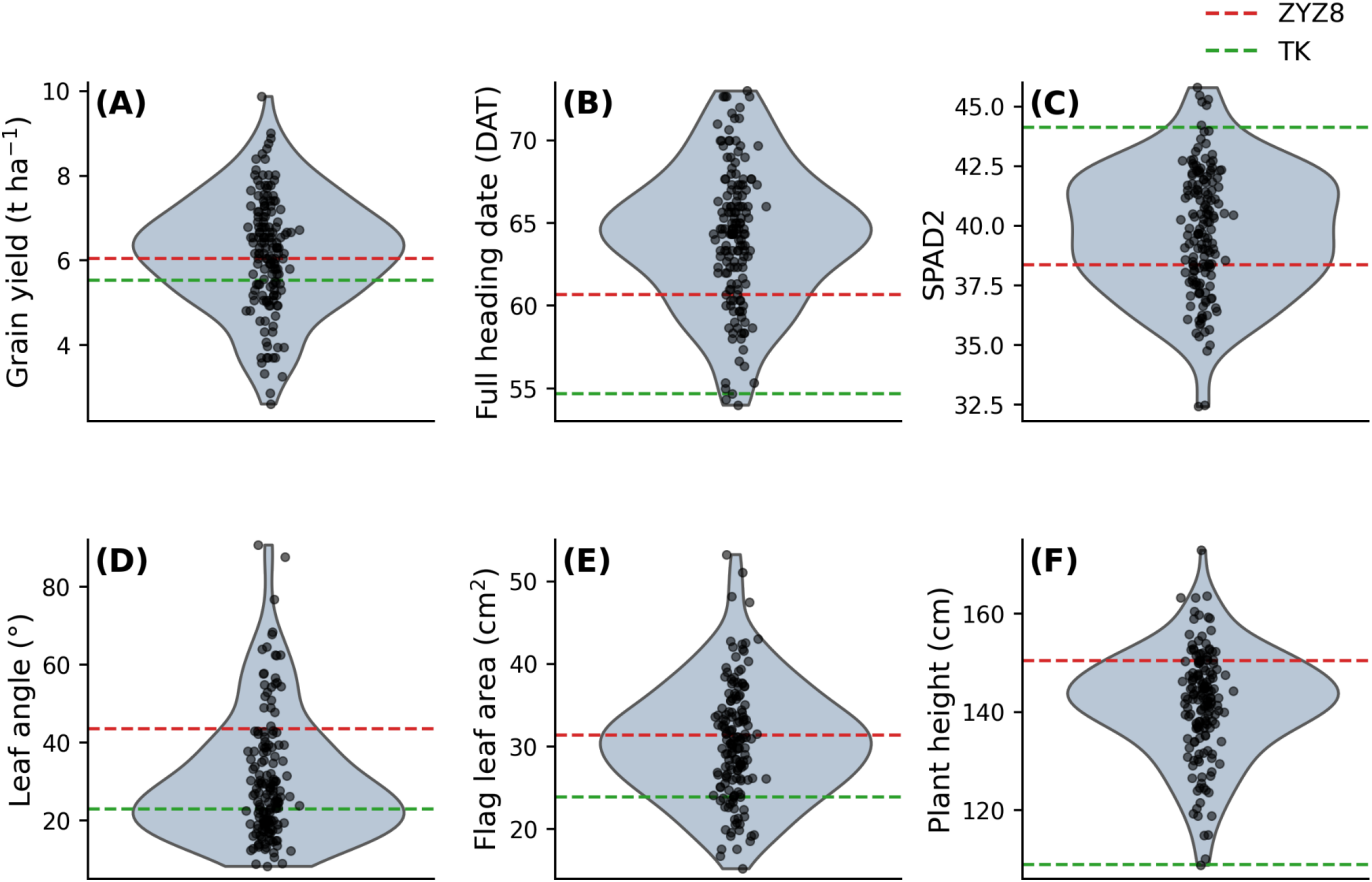
Phenotypic distributions of selected traits in the *indica–japonica* RIL population. Violin plots show distributions of 159 RILs based on means across three replicates: **(A)** grain yield, **(B)** full heading date, **(C)** SPAD2, **(D)** flag leaf angle, **(E)** flag leaf area, and **(F)** plant height. Horizontal dashed red and green lines indicate the parental values of ZYZ8 and TK, respectively.

Representative phenotypic distributions of grain yield, full heading date, post-heading SPAD at the onset of grain filling (SPAD2), flag leaf angle, flag leaf area, and plant height are shown in **Figure 1**. All six traits exhibited continuous distributions across the RIL population, consistent with quantitative inheritance. The two parents differed in several key traits, whereas the RIL population extended beyond the parental range for grain yield, heading-related traits, and leaf architectural traits, indicating evident transgressive segregation. Together, these results demonstrate that the *indica–japonica* RIL population possessed abundant phenotypic diversity and a strong genetic basis for subsequent analyses of trait relationships, multivariate phenotypic patterns, and promising line selection.

### 3.2 Phenotypic associations of grain yield with heading progression, flag-leaf SPAD dynamics, and upper-leaf architecture

**Figure 2:**
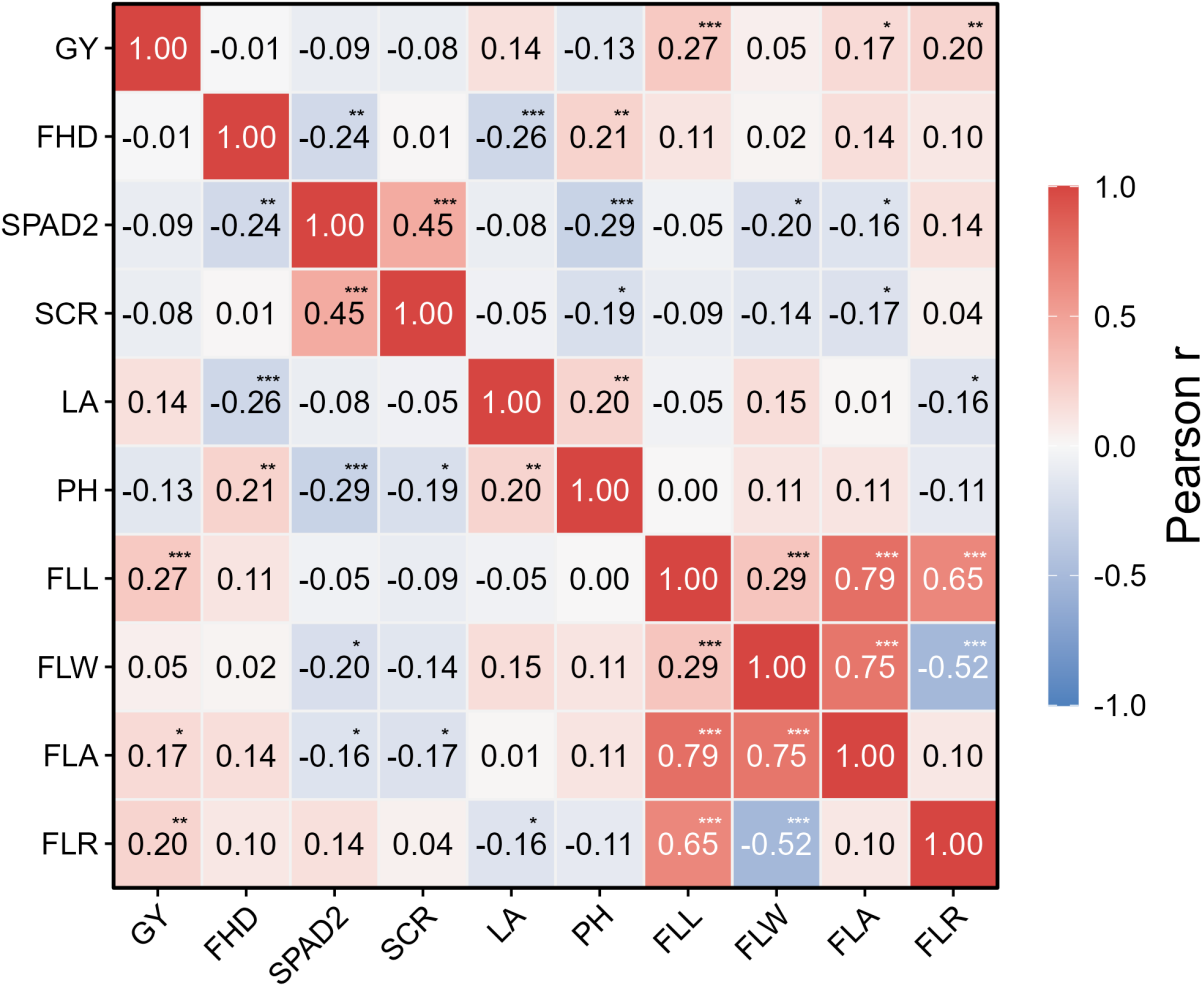
Pearson correlation matrix among grain yield and representative phenotypic traits in the *indica–japonica* RIL population. Values indicate Pearson correlation coefficients based on genotype means. Significance levels are indicated as *P < 0.05, **P < 0.01, and ***P < 0.001. Because the correlation matrix was used for exploratory description of phenotypic associations, P values were not adjusted for multiple testing. GY, grain yield; FHD, full heading date; SPAD2, post-heading SPAD at the onset of grain filling; SCR, SPAD change rate; LA, flag leaf angle; PH, plant height; FLL, flag leaf length; FLW, flag leaf width; FLA, flag leaf area; FLR, flag leaf length/width ratio.

Pearson’s correlation analysis was used to describe exploratory phenotypic associations between grain yield and the measured trait modules (**Figure 2**). Among the evaluated traits, flag leaf length showed the strongest positive association with grain yield (r = 0.270, P = 0.001), followed by flag leaf length/width ratio (r = 0.204, P = 0.010) and flag leaf area (r = 0.170, P = 0.033). Flag leaf angle was positively associated with grain yield at a marginal level (r = 0.135, P = 0.089). In contrast, full heading date, SPAD2, SPAD change rate, and plant height showed weak or non-significant associations with grain yield.

The correlation structure further indicated that traits within the same phenotypic module were closely related. In particular, flag leaf area was strongly associated with flag leaf length and width, whereas SPAD-related indices were more closely linked to one another than to heading-related traits. These patterns suggested that upper-leaf architectural traits, especially those reflecting flag leaf elongation and shape, were associated with grain yield variation under the conditions evaluated, but the observed associations should not be interpreted as direct causal effects.

To further identify the major phenotypic variables associated with grain yield, a multiple linear regression model was fitted using one representative variable from each trait module (**Table 2**). Collinearity among explanatory variables was low, with all variance inflation factor values below 1.2. The final model explained 0.132 of the variation in grain yield (R² = 0.132, adjusted R² = 0.103; P < 0.001), indicating modest explanatory power. Among the included predictors, flag leaf length showed a positive association with grain yield (standardized β = 0.266; P = 0.001), flag leaf angle also showed a positive association (standardized β = 0.199; P = 0.015), and plant height showed a negative association (standardized β = −0.203; P = 0.014). Full heading date and SPAD change rate were not significant in the final model. Together, these results indicated that leaf and architecture-related traits accounted for only a limited proportion of yield variability. Additional unmeasured yield components may also have contributed substantially to grain yield variation.

**Table 2:** Multiple linear regression model for grain yield in the *indica–japonica* RIL population, including standardized regression coefficients.

Variable	Estimate	SE	t value	P value	Std. β
Intercept	5.831	1.978	2.948	0.004	—
FHD	0.018	0.026	0.706	0.481	0.058
SCR	-0.019	0.017	-1.117	0.266	-0.086
LA	0.017	0.007	2.454	0.015	0.199
PH	-0.025	0.010	-2.474	0.014	-0.203
FLL	0.088	0.025	3.487	0.001	0.266

FHD, full heading date; SCR, SPAD change rate; LA, flag leaf angle; PH, plant height; FLL, flag leaf length; β, standardized regression coefficient calculated after z-score standardization of grain yield and explanatory variables. The final model included FHD, SCR, LA, PH, and FLL as explanatory variables; R² = 0.132, adjusted R² = 0.103, and overall model P < 0.001.

### 3.3 Multivariate phenotypic patterns associated with grain yield variation

Principal component analysis (PCA) was performed using six representative variables to summarize multivariate phenotypic variation in the *indica–japonica* RIL population. These variables were selected to represent the three phenotypic modules emphasized in this study while reducing redundancy among correlated measurements: initial heading date (IHD), post-heading SPAD at the onset of grain filling (SPAD2), SPAD change rate (SCR), flag leaf length (FLL), flag leaf area (FLA), and flag leaf length/width ratio (FLR). The first four principal components explained 67.0% of the total phenotypic variance, with PC1, PC2, PC3, and PC4 accounting for 21.4%, 17.5%, 15.1%, and 13.0%, respectively (**Table 3**). These results indicated that yield-related phenotypic variation in the RIL population was jointly associated with multiple trait dimensions rather than with a single dominant trait.

**Table 3:** Explained variance and dominant trait loadings of the first four principal components.

Principal component	Explained variance (%)	Cumulative variance (%)	Dominant trait loadings (top 3 by absolute value)
PC1	21.4	21.4	FLL (+), FLA (+), FLR (+)
PC2	17.5	38.9	SPAD2 (+), SCR (+), FLR (+)
PC3	15.1	54.0	IHD (+), SCR (+), FLA (−)
PC4	13.0	67.0	FLR (+), FLA (−), SCR (−)

IHD, initial heading date; SPAD2, post-heading SPAD at the onset of grain filling; SCR, SPAD change rate; FLL, flag leaf length; FLA, flag leaf area; FLR, flag leaf length/width ratio. Dominant trait loadings are the top three loadings by absolute value.

The PCA loading matrix further showed that different principal components captured distinct phenotypic modules. PC1 was mainly associated with upper-leaf morphology, with major positive loadings from flag leaf length, flag leaf area, and flag leaf length/width ratio. PC2 was primarily associated with post-heading SPAD status and SPAD change rate, together with flag leaf length/width ratio. PC3 was dominated by initial heading date, SPAD change rate, and flag leaf area, whereas PC4 was mainly driven by flag leaf length/width ratio, flag leaf area, and SPAD change rate (**Table 3**).

**Figure 3:**
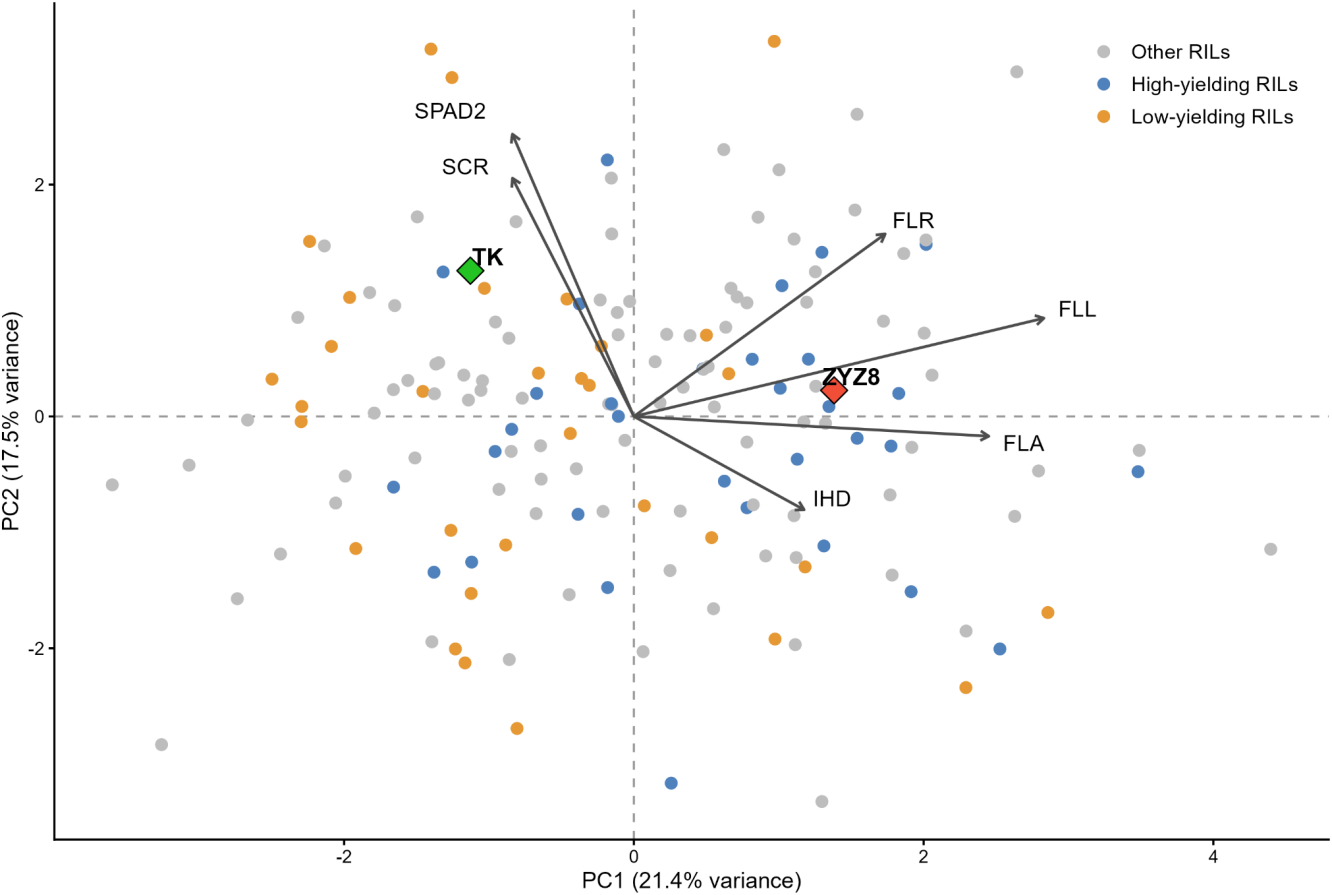
PCA biplot of yield-related phenotypic traits in the *indica–japonica* RIL population. Gray points indicate the remaining RILs, blue points indicate high-yielding RILs, and orange points indicate low-yielding RILs. Diamond symbols represent the parental lines. Arrows indicate selected trait loadings for initial heading date (IHD), post-heading SPAD at the onset of grain filling (SPAD2), SPAD change rate (SCR), flag leaf length (FLL), flag leaf area (FLA), and flag leaf length/width ratio (FLR).

The PCA biplot based on PC1 and PC2 is shown in **Figure 3**. Although the high- and low-yielding lines were not completely separated in the two-dimensional space, high-yielding lines tended to be distributed toward the positive side of PC1, whereas many low-yielding lines were located toward the negative side of PC1. The two parents were positioned outside the central bulk of the RIL population, further illustrating the broad phenotypic recombination generated in the derived lines.

Overall, the PCA results supported the view that phenotypic variation related to grain yield in this population was associated chiefly with upper-leaf morphology and SPAD dynamics, with heading progression contributing an additional but smaller dimension. This multivariate pattern provided a basis for subsequent comparison between high-yielding and low-yielding groups and for the preliminary identification of candidate lines with favorable phenotypic combinations.

### 3.4 Comparison of phenotypic traits between high-yielding and low-yielding groups

**Figure 4:**
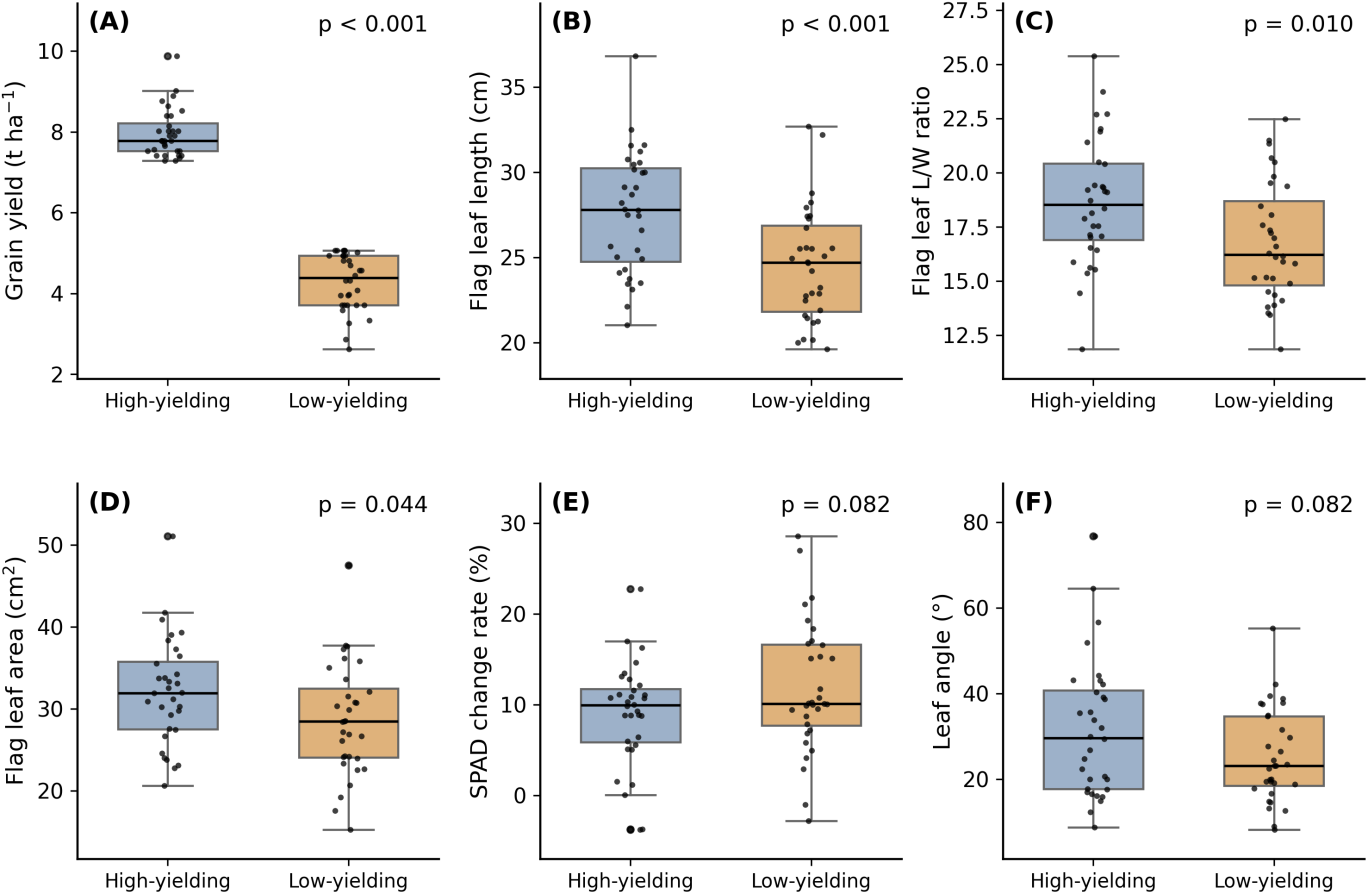
Comparison of key traits between high-yielding and low-yielding groups: **(A)** grain yield, **(B)** flag leaf length, **(C)** flag leaf length/width ratio, **(D)** flag leaf area, **(E)** SPAD change rate, and **(F)** flag leaf angle. Asterisks indicate significant differences between groups.

The 159 RILs were ranked according to mean grain yield across the three replicates, and the top 20% (32 lines) and bottom 20% (32 lines) were defined as the high-yielding and low-yielding groups, respectively. Comparison between the two groups revealed a pronounced yield contrast, with mean grain yield reaching 7.96 t ha^-^¹ in the high-yielding group and 4.27 t ha^-^¹ in the low-yielding group (P < 0.001; **Table 4**; **Figure 4**).

**Table 4:** Comparison of phenotypic traits between the high-yielding and low-yielding groups in the *indica–japonica* RIL population.

Trait	High-yielding group	Low-yielding group	Difference	P-value
Grain yield	7.960	4.273	3.687	<0.001
Flag leaf length	27.637	24.567	3.071	<0.001
Flag leaf length/width ratio	18.664	16.796	1.869	0.010
Flag leaf area	32.080	28.629	3.451	0.044
SPAD change	3.005	4.008	-1.003	0.075
SPAD change rate	8.957	11.828	-2.871	0.082
Flag leaf angle	31.519	25.452	6.067	0.082
SPAD2	38.759	39.762	-1.004	0.165
Plant height	139.698	143.771	-4.073	0.169
Heading interval	7.198	6.917	0.281	0.441
Flag leaf width	1.508	1.485	0.023	0.688
Initial heading date	57.406	57.802	-0.396	0.692
Full heading date	64.604	64.719	-0.115	0.918
SPAD1	35.754	35.754	-0.000	0.999

Values are genotype means of the top 20% (32 lines) and bottom 20% (32 lines) of the RIL population ranked by grain yield. P values were obtained using Welch’s two-sample t-test. SPAD1, booting-stage SPAD before heading onset; SPAD2, post-heading SPAD at the onset of grain filling.

Among the measured traits, the high-yielding group exhibited significantly greater flag leaf length, flag leaf length/width ratio, and flag leaf area than the low-yielding group (P < 0.05; **Table 4**). In addition, the high-yielding group tended to show a smaller SPAD change, a lower SPAD change rate, and a larger flag leaf angle, although these differences were only marginally significant (P = 0.075, 0.082, and 0.082, respectively).

In contrast, heading-related traits, including initial heading date, full heading date, and heading interval, did not differ significantly between the two groups. SPAD1 and SPAD2 also showed no significant difference, and plant height only exhibited a weak decreasing trend in the high-yielding group. Taken together, these results indicated that higher-yielding lines were characterized less by differences in heading time and more by favorable upper-leaf morphology and relatively stable SPAD dynamics.

### 3.5 Identification of preliminary candidate lines with favorable phenotypic combinations

Based on grain yield and multivariate phenotypic performance, preliminary candidate RILs were further identified for future validation. The 159 RILs were first ranked according to mean grain yield across the three replicates, and the top 20% of lines were then prioritized for further screening. Within this high-yielding subset, a composite score was calculated from standardized values of grain yield (GY), flag leaf length (FLL), flag leaf length/width ratio (FLR), leaf angle (LA), inverse plant height (−PH), and inverse SPAD change rate (−SCR), and the resulting score was used to rank lines with favorable phenotypic combinations (**Table 5**). Because this procedure was based on a single field environment and grain yield was included in both the initial selection and the composite score, the selected lines should be interpreted as preliminary candidates rather than independently validated stable high-yielding lines.

**Table 5:** Preliminary candidate high-yielding RILs identified within the top-yielding subset by composite score under a single field environment.

Rank	RIL	GY (t ha^-^¹)	FLL (cm)	FLR	LA (°)	PH (cm)	SCR (%)	Composite score
1	RIL143	7.53	31.24	23.74	42.15	146.58	-3.75	0.712
2	RIL072	8.02	32.50	25.38	33.83	132.72	10.85	0.707
3	RIL149	8.64	31.58	17.53	64.56	140.57	8.82	0.645
4	RIL050	8.02	36.83	17.53	56.61	148.09	5.56	0.612
5	RIL114	8.52	30.01	22.71	17.63	120.32	11.06	0.597
6	RIL083	9.88	30.58	19.36	15.94	137.75	12.14	0.489
7	RIL047	8.89	29.10	20.48	44.22	135.25	14.66	0.467
8	RIL113	7.28	27.76	22.03	14.99	110.09	6.44	0.376
9	RIL109	8.40	30.46	22.68	40.33	150.31	11.59	0.331
10	RIL081	7.78	29.12	21.41	51.91	138.03	13.49	0.282

Composite score was calculated as described in Section 3.5. These lines should be regarded as preliminary candidates from a single-environment phenotypic screening rather than independently validated stable high-yielding lines. GY, grain yield; FLL, flag leaf length; FLR, flag leaf length/width ratio; LA, flag leaf angle; PH, plant height; SCR, SPAD change rate.

The selected preliminary candidate lines generally combined higher grain yield with longer flag leaves, larger flag leaf length/width ratios, and favorable upper-leaf architecture. Compared with the population means, the top 10 selected lines exhibited higher mean grain yield (8.30 vs. 6.18 t ha^-^¹), longer flag leaves (30.92 vs. 26.35 cm), larger flag leaf area (33.95 vs. 30.51 cm²), greater flag leaf length/width ratio (21.29 vs. 18.00), and larger flag leaf angle (38.22 vs. 30.53°). They also showed a generally smaller SPAD change rate than the population mean (mean SCR = 9.09% vs. 9.48%) and moderately reduced plant height (135.97 vs. 141.64 cm). These lines provide useful materials for subsequent multi-environment testing and genetic analysis, but their yield stability remains to be validated.

## 4 Discussion

### 4.1 Grain yield variation in the RIL population was associated with upper-leaf architecture under the conditions evaluated

In the present single-environment field evaluation of an *indica–japonica* RIL population, upper-leaf architecture was associated with grain yield variation under the conditions evaluated, whereas heading progression itself showed a weaker relationship with yield (**Figures 2**, **4**; **Tables 2**,**4**). SPAD dynamics provided complementary but weaker information on coordinated phenotypic performance. Although heading-related traits showed substantial phenotypic variation and relatively high heritability (**Table 1**), neither initial heading date, full heading date, nor heading interval differed significantly between the high-yielding and low-yielding groups (**Table 4**). This result suggests that, under the field conditions of the present study, heading progression per se was not the main phenotypic factor associated with yield differences among lines. Such a pattern is consistent with previous studies showing that heading date contributes to rice productivity mainly by placing genotypes within an ecologically suitable developmental window, rather than by conferring a simple yield advantage through delayed heading alone (Yano et al., 2000; Xue et al., 2008; Sun et al., 2023; Su et al., 2025). It is also consistent with reports that heading-date loci often show pleiotropic effects on plant height and other agronomic traits, which complicates the direct interpretation of heading time per se as a yield determinant (Zhou et al., 2016; Lee et al., 2024).

By contrast, several upper-leaf traits showed clearer phenotypic associations with grain yield (**Figure 2**; **Table 2**). Flag leaf length, flag leaf area, and flag leaf length/width ratio were positively associated with grain yield, and the high-yielding group exhibited significantly longer flag leaves, larger flag leaf area, and a greater length/width ratio than the low-yielding group (**Table 4**). These results agree with previous studies showing that flag leaf morphology contributes substantially to source capacity, canopy photosynthesis, and yield-related performance in rice (Wang et al., 2011; Zhang et al., 2014; Fabre et al., 2016; Wang et al., 2023; Singh et al., 2024). Recent synthesis centered on NAL1 further highlights the importance of leaf architecture for coordinating source–sink relations and yield realization in rice (Velu et al., 2025). Nevertheless, the regression model had modest explanatory power, indicating that leaf traits explained only a limited extent of yield variability. Therefore, additional yield components and other unmeasured factors likely contributed to the observed variation in grain yield.

SPAD dynamics around heading also provided complementary, though weaker, information on phenotypic performance (**Figure 2**; **Table 4**). High-yielding lines tended to show a smaller SPAD change and lower SPAD change rate between SPAD1 and SPAD2, indicating more stable flag-leaf chlorophyll status across the transition from pre-heading to early grain filling. However, the correlations involving SPAD change and the differences between high- and low-yielding groups were not strong, and these traits should therefore be interpreted as supplementary indicators rather than primary determinants of yield. This tendency agrees with previous studies showing that reproductive-stage leaf color, chlorophyll dynamics, and flag-leaf photosynthetic traits are associated with grain filling, nitrogen remobilization, and late-stage nitrogen reuse in rice (Kim and Kim, 2024; Tao et al., 2024; Xu et al., 2025; Riaz et al., 2026). It also agrees with the broader concept of crop “physiotype,” which emphasizes sustained source activity during reproductive growth as one component of final productivity (Li et al., 2025b; Wang et al., 2025; Yagioka et al., 2025).

Flag leaf angle showed a positive tendency with grain yield and contributed significantly in the regression model (**Table 2**), although its difference between the high- and low-yielding groups was only marginally significant (**Table 4**). This result should be interpreted together with flag leaf size rather than as an isolated effect. Flag leaf angle can modify light penetration and the within-canopy light environment (Burgess et al., 2017; Huang et al., 2021), but its effect on yield depends on how it is coordinated with other architectural traits and with overall canopy configuration (Li et al., 2025a; Zhu et al., 2020). Taken together, these results indicate that the favorable phenotype associated with higher yield in this population was characterized less by variation in heading progression itself and more by a coordinated combination of flag-leaf SPAD dynamics, flag leaf morphology, and plant architecture. A similar integrative interpretation has been reported in physiological analyses of high-yielding rice, in which stable productivity was associated with coordinated morphological and physiological trait combinations rather than with a single dominant trait (Pan et al., 2023; Yagioka et al., 2025; Yin et al., 2021).

### 4.2 Coordinated phenotypic combinations were associated with higher yield performance and provided hypotheses for breeding selection

The multivariate analyses further indicated that higher yield performance in this population was associated with coordinated phenotypic combinations involving upper-leaf morphology and, to a lesser extent, SPAD dynamics and heading progression (**Figure 3**; **Tables 4**, **5**). The PCA did not completely separate the high- and low-yielding lines in two-dimensional space (**Figure 3**), but it captured major dimensions of variation related to upper-leaf morphology, SPAD dynamics, and heading progression (**Table 3**). Likewise, the preliminary candidate lines identified within the high-yielding subset were not distinguished by grain yield alone; rather, they combined relatively strong yield performance with longer flag leaves, greater flag leaf length/width ratio, favorable leaf angle, and lower SPAD change rates (**Table 5**). Because these candidates were identified from a single environment and from an exploratory phenotypic screening procedure, they should be considered hypotheses for subsequent validation rather than confirmed selection criteria. This interpretation is consistent with ideotype breeding concepts in rice, which emphasize that productivity emerges from coordinated combinations of architecture, source activity, and developmental timing rather than from any single trait considered alone (Peng et al., 2008; Li et al., 2025b; Wang et al., 2025). It also agrees with physiological analyses of high-yielding rice cultivars, in which productivity is associated with coordinated trait combinations rather than with a single dominant feature (Pan et al., 2023; Yagioka et al., 2025).

This coordinated-trait perspective is particularly relevant for *indica–japonica* populations. The *indica* and *japonica* subspecies harbor substantial genetic divergence and complementary favorable alleles (Marathi et al., 2012; Bai et al., 2011; Zhang et al., 2014), and recombination between them can generate broad phenotypic segregation and transgressive variation in derived lines (Bai et al., 2011; Zhang et al., 2014; Hu et al., 2025). In the present study, continuous trait distributions together with evident transgressive segregation (**Figure 1**; **Table 1**) indicate that the population provided substantial scope for phenotypic selection. Such patterns are consistent with previous reports that inter-subspecific recombination can create novel combinations of developmental, architectural, and yield-related traits that extend beyond the parental range and thereby increase breeding potential (Marathi et al., 2012; Bai et al., 2011; Zhang et al., 2014).

From a breeding perspective, the present results suggest that selection in this population should focus on coordinated phenotypic performance rather than on individual traits in isolation. In the present study, upper-leaf morphology provided clearer discrimination between high- and low-performing lines than heading-related traits alone, while SPAD dynamics between SPAD1 and SPAD2 provided complementary information on phenotypic coordination (**Figure 2**; **Table 4**). This does not imply that heading date is unimportant, but rather that, once a population falls within a broadly suitable phenological range, selection efficiency may be improved by giving greater weight to traits reflecting canopy structure and SPAD dynamics across the pre-heading-to-early-grain-filling transition. This interpretation is consistent with recent efforts to optimize rice productivity by combining favorable developmental windows with trait combinations that enhance resource capture, reproductive-stage performance, and nitrogen-responsive flowering stability (Su et al., 2025; Yoshida et al., 2025; Li et al., 2025b; Takai et al., 2013; Yagioka et al., 2025). Therefore, the lines identified here should be regarded as preliminary candidates from a single-environment phenotypic screening, rather than as confirmed stable high-yielding lines. Their value lies in carrying favorable combinations of grain yield, flag-leaf morphology, plant architecture, and SPAD-related profiles that warrant further multi-environment validation (**Table 5**).

### 4.3 Limitations of the present study and implications for future research

Several limitations of the present study should be acknowledged. First, the current analysis was conducted in a single field environment and within one growing season. Although this design was sufficient to detect substantial phenotypic segregation and to identify informative trait combinations associated with grain yield, the stability of these relationships across environments remains to be tested. Second, the materials were arranged in numerical order within each block rather than randomly assigned to facilitate line identification and reduce phenotyping and harvest errors. This layout may introduce potential field-position effects within blocks, and such effects cannot be completely excluded despite retaining block effects in the statistical analysis. Therefore, the observed trait-yield relationships should be interpreted cautiously as phenotypic associations under the present experimental conditions. Third, detailed yield components, including panicle number, spikelets per panicle, seed-setting rate, and thousand-grain weight, were not measured. This was because the present study was designed as a population-level field phenotyping study centered on plot-level grain yield and non-destructive upper-leaf measurements, rather than a full component dissection of yield formation. These unmeasured yield components may have contributed substantially to yield variability and should be included in future validation. Fourth, the present study did not incorporate a complete genetic dissection of the observed phenotypic associations, which has been informative in QTL- and gene-focused studies of rice yield variation (Bai et al., 2011; Marathi et al., 2012; Li et al., 2023). Accordingly, the present work should be interpreted as an exploratory population-level phenotypic dissection rather than as a mechanistic analysis of yield formation.

Despite these limitations, the present study provides a useful basis for future work. By showing that grain yield variation in this population was associated more strongly with upper-leaf architecture than with heading progression alone under the conditions evaluated, while SPAD dynamics supplied complementary information on coordinated phenotypic performance (**Figures 2**, **4**; **Table 5**), the study narrows the range of traits that merit priority in subsequent validation and genetic analysis. Future studies should determine whether the favorable phenotypic combinations identified here remain stable across environments, whether they are associated with specific yield-component patterns, and whether they can be linked to stable genomic regions or known heading-date and yield-related loci, as reported in earlier genetic studies (Bai et al., 2011; Marathi et al., 2012; Sun et al., 2023; Li et al., 2023). Overall, these findings are exploratory and require multi-environment validation before the identified trait combinations or candidate lines can be used as stable selection criteria in breeding. In this sense, the current work provides a set of preliminary candidate materials and a phenotypic framework for subsequent validation and future genetic dissection of favorable trait combinations associated with grain yield.

## Data Availability

The raw data supporting the conclusions of this article will be made available by the authors, without undue reservation.
